# Editorial: Highlights from the Botrytis and Sclerotinia 2022 Joint Conference

**DOI:** 10.3389/fpls.2023.1326020

**Published:** 2023-11-08

**Authors:** Maya Bar, Gianfranco Romanazzi

**Affiliations:** ^1^ Department of Plant Pathology and Weed Research, Agricultural Research Organization (ARO), Rishon LeZion, Israel; ^2^ Department of Agricultural, Food and Environmental Sciences, Marche Polytechnic University, Ancona, Italy

**Keywords:** *Botrytis*, *Sclerotinia*, fungal diversity, host adaptation, fungal development, fungal epidemiology, disease management, host pathogen interaction

*Botrytis* spp. and *Sclerotinia* spp. are fungal plant pathogens of major agricultural importance. These related fungi, both of the family *Sclerotiniaceae*, target many economically important crops, and a lot of fungicides are currently employed worldwide in an effort to control them (Williamson et al., 2007; Fillinger and Elad, 2016). Urgent interventions are required to better understand these pathogens and mitigate their effects on crop production. This Research Topic aimed to bring together new biological research and technical innovations concerning these pathogens and their interactions with plants, to ultimately help the development of advanced effective and sustainable control methods.

The international Botrytis conference has been bringing together researchers investigating all aspects of Botrytis biology since 1966. The conference has been held every 2-4 years since 1966, and is the central conference for *Botrytis* researchers worldwide. In a similar way, the *Sclerotinia* workshop, held roughly every 3-4 years since 1974, has been dedicated to all aspects of *Sclerotinia* research.

Prior to the 18^th^ BotrySclero conference, researchers collaborating in both fields had decided to unify the *Botrytis* and *Sclerotinia* events, given the similarity of these fungi and their host ranges (De Miccolis Angelini et al., 2022), and the fact that many research groups study both fungi, creating a significant overlap in the attendees of both events. Continuing with this inclusive theme of related fungal phytopathogen species, the upcoming conference to be held in Thessaloniki, Greece, in 2025, will include *Monilinia* researchers and be dubbed ‘BotryScleroMoni’.

Postponed from 2020 due to COVID-19, the BotrySclero conference, which was also the 18^th^ *Botrytis* conference and the 17^th^ *Sclerotinia* workshop, was held in June of 2022 in Avignon, France, after a successful online conference promoting early career scientists in this field was held in 2021. Thus, the excellent organizing committee in practice organized two separate conferences. The online conference constituted a significant contribution to researchers in the field who were not able to meet in person due to COVID-19 restrictions.

During the 2022 conference, which for many of us was the first “real” conference we attended following the COVID-19 pandemic, leading researchers in the *Botrytis* and *Sclerotinia* fields presented work categorized in several topics including fungal diversity, host adaptation, and development; -omic methods in *Botrytis* and *Sclerotinia* research; ecology and epidemiology of these fungi; host-pathogen interactions, and disease management. Uniquely, this conference continues over the years to bring together individuals engaged in both basic and applied research projects. Thus, basic themes of fungal development, host adaptation and ecological aspects of fungal biology were presented and contemplated by all present, alongside applicable aspects relating to the epidemiology and management of gray mold (caused by *Botrytis*) and white rot (caused by *Sclerotinia*), both pre and postharvest, as well as aspects relating to the noble wine production industry, where Botrytis is used as part of the production process, owing to berry dehydration that increases sugar content (Magyar, 2011).

The innovative research presented at the conference can be divided into several themes, including Fungal development and Functional genomics (Fall et al., 2018; Hahn and Scalliet, 2021; Henríquez-Urrutia et al., 2022; Schumacher, 2023); Host adaptation and specificity (Clarkson et al., 2017; Liang and Rollins, 2018; de Vallée et al., 2019; Lacrampe et al., 2021; Mercier et al., 2021; Michael et al., 2021; Silva et al., 2021a; Silva et al., 2021b; You and van Kan, 2021; Anand et al., 2022; Rombach et al.), virulence mechanisms, including mycoviruses (Kamaruzzaman et al., 2019; Fu et al., 2023), bioactive peptides, extracellular vesicles (Souibgui et al., 2021; De Vallée et al., 2023), and secretion (Lyu et al., 2016; Xie et al., 2021); Epidemiology, Ecology, and disease management (Pintye et al., 2015; Nicolaisen et al., 2017; Chen et al., 2018; Fall et al., 2018; Wytinck et al., 2020; Samaras et al., 2021; Romanazzi and Moumni, 2022); and host adaptation, virulence, and disease management aspects related to wine grapes and noble wine (Hegyi et al., 2022; Mundy et al., 2022; Jiang et al., 2023). Notably, the use of cutting edge technologies for both genetic and -omic analyses, and disease detection and management, was evident in the research presented.

In line with these diverse and multi-disciplinary aspects of *Botrytis* and *Sclerotinia* research, the Research Topic entitled “*Highlights from the Botrytis and Sclerotinia 2022 Joint Conference*” includes 6 research works, relating mainly to host pathogen interactions and disease control.

In Rombach et al., the interaction between *B. cinerea* and its tomato host is examined in the context of the under-investigated response of the fungus to the tomato leaf microstructure, using cutting-edge biomimetics. The authors found that *B. cinerea* spore distribution differs based on surface microstructure, leading to a structure-based germination response.

Qin et al. examined the possible functionality of plant small RNAs in down-regulating *B. cinerea* virulence by silencing fungal virulence genes through a cross-kingdom RNAi process, finding that a particular tomato sRNA potentially targeting a fungal virulence gene was actually not responsible for fungal virulence gene down-regulation.

Gupta et al. investigated viral-fungal co-infection using *B. cinerea* and the agriculturally devastating tobamovirus ToBRFV. Such co-infections are becoming increasingly relevant, especially when several sub-lethal pathogens are present simultaneously, a situation which has been suggested to be exacerbated by climate change. The authors found that tobamovirus-mediated accumulation of salicylic acid increases the plants’ susceptibility to *B. cinerea*, suggesting that ToBRFV may pose additional risks in agriculture.

In a cautionary work, You et al. investigated the basis of *B. cinerea* resistance in the wild tomato relative *Solanum habrochaites*, finding that inoculation methods and media used in laboratory settings can affect disease assay outcomes, with either a low spore density or a high sugar concentration in the inoculum abolishing the potential disease resistance of tomato hosts.

Sofianos et al. addressed another important issue in gray mold management - the ever present risk of emergence of newly fungicide resistant isolates. Characterizing the genetic basis for fungicide resistance in isolates from strawberries, rootstocks, and tomatoes, their work shows that mutations leading to multiple resistance or multidrug resistance are highly prevalent, underscoring the importance of resistance management in different crops.

Finally, Altieri et al. provide a perspective on the efficacy of different commercial biocontrol agents in reducing gray mold in grapevine, as compared with a commercial fungicide. Their wide data analysis confirms that biocontrol efficacy is highly environmentally sensitive, supporting the notion that current research into biocontrol efficacy should focus on environmental interactions.

Overall, these 6 papers demonstrate the varied research the *Botrytis* and *Sclerotinia* communities are engaged in, and provide some of the open questions which will likely be addressed in the upcoming conference in 2025.

## Author contributions

MB: Conceptualization, Writing – original draft, Writing – review & editing. GR: Conceptualization, Writing – review & editing.
